# Epiviz: a view inside the design of an integrated visual analysis software for genomics

**DOI:** 10.1186/1471-2105-16-S11-S4

**Published:** 2015-08-13

**Authors:** Florin Chelaru, Héctor Corrada Bravo

**Affiliations:** 1 Center for Bioinformatics and Computational Biology, University of Maryland, College Park, MD, USA; 2 Department of Computer Science, University of Maryland, College Park, MD, USA

**Keywords:** Visualization, Functional Genomics, DNA Methylation

## Abstract

**Background:**

Computational and visual data analysis for genomics has traditionally involved a combination of tools and resources, of which the most ubiquitous consist of genome browsers, focused mainly on integrative visualization of large numbers of big datasets, and computational environments, focused on data modeling of a small number of moderately sized datasets. Workflows that involve the integration and exploration of multiple heterogeneous data sources, small and large, public and user specific have been poorly addressed by these tools. In our previous work, we introduced Epiviz, which bridges the gap between the two types of tools, simplifying these workflows.

**Results:**

In this paper we expand on the design decisions behind Epiviz, and introduce a series of new advanced features that further support the type of interactive exploratory workflow we have targeted. We discuss three ways in which Epiviz advances the field of genomic data analysis: 1) it brings code to interactive visualizations at various different levels; 2) takes the first steps in the direction of collaborative data analysis by incorporating user plugins from source control providers, as well as by allowing analysis states to be shared among the scientific community; 3) combines established analysis features that have never before been available simultaneously in a genome browser. In our discussion section, we present security implications of the current design, as well as a series of limitations and future research steps.

**Conclusions:**

Since many of the design choices of Epiviz are novel in genomics data analysis, this paper serves both as a document of our own approaches with lessons learned, as well as a start point for future efforts in the same direction for the genomics community.

## Background

We provide an insight into the design choices of Epiviz [1], a tool for interactive visual and computational analysis of genomic data. We include design choices behind the tool and introduce new features that greatly broaden our support for interactive analysis workflows of genomic data over our previously published general overview of its architecture and design. We present a use case of epigenomic data analysis that makes use of the improvements of Epiviz based on these new extensions and features.

### Motivation

The design of Epiviz responds to the need of integrating computational and visual interactive and exploratory analysis for genomics data. Existing tools usually treat these steps: 1) computational and statistical analysis, and 2) interactive integrative visualization, as distinct (Sup. Table 1 in Additional file 1), while they are more effective when used iteratively.

**Figure 1:**
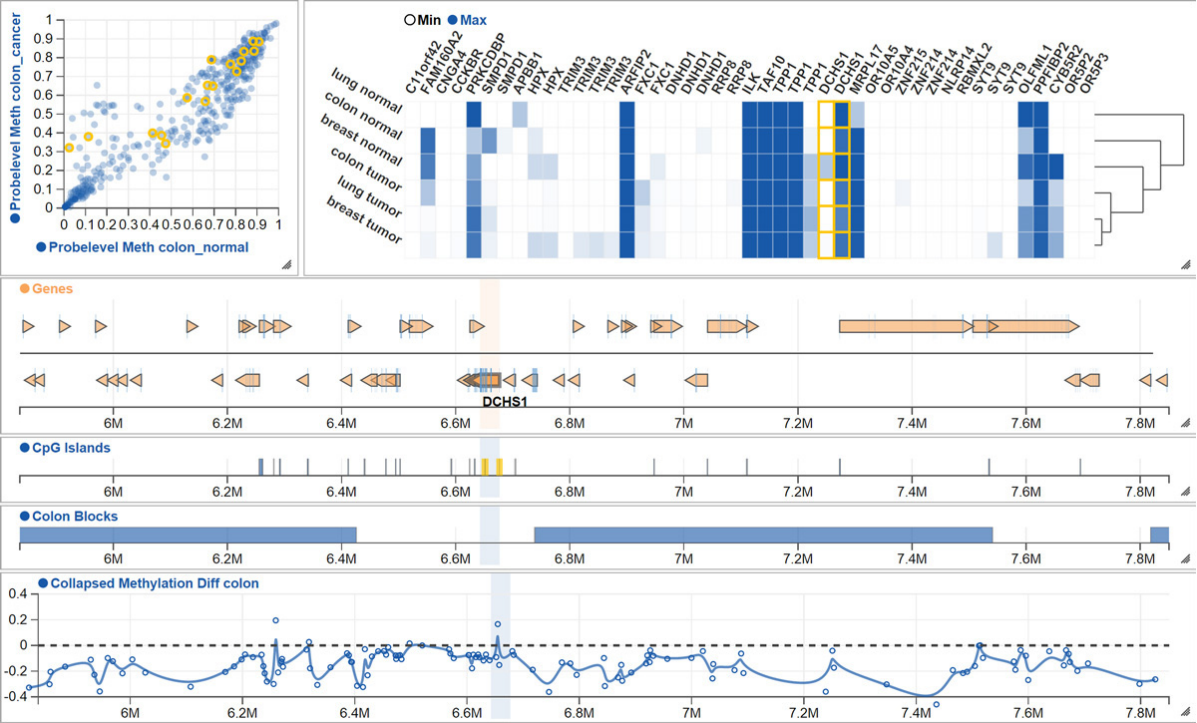
**Statistical analysis of colon cancer methylome**. Top-left displays CpG-level methylation measurements for colon normal and tumor tissue for a sample of TCGA [12] data; top-right displays expression data from the Gene Expression Barcode Project across multiple cancer types as a *heatmap*, the hierarchical clustering is dynamically updated as the users navigates across the genome; the bottom display shows smooth function $g_{k}\left(l\right)$, described in the main text, corresponding to differences in methylation between normal and tumor; the "Colon Blocks" track displays statistically significant DMRs inferred from the smoothed methylation difference function. The brushing interaction (in yellow) links data from Bioconductor objects produced at different stages of a statistical analysis pipeline: from measurement preprocessing to statistically significant regions of interest allowing effective exploration of the statistical properties of these genomic findings. http://epiviz.cbcb.umd.edu/?ws=sp9ShCJdS3c

Consider the case of finding differentially methylated regions (DMRs) associated with cancer. Statistical inferences from smoothing methods built on base-pair level measurements of DNA methylation (DNAm) [2,3] are used to carry out this task. In a general sense, these methods use a statistical model of expected methylation $m_{ljk}$ at genomic locus *l *for replicate *j *belonging to class *k *(say, normal or tumor): $m_{ljk}=f\left(l\right)+g_{k}\left(l\right)+e_{ljk}$ where $f\left(l\right)$ is a smooth function over genomic loci and $g_{k}\left(l\right)$ is another smooth function that measures deviation in methylation for class *k*, so that contiguous regions where $\left|g_{k}\left(l\right)\right|$ is large enough are considered differentially methylated and a level of significance can be determined by parametric or non-parametric statistical methods. This stipulates a chain of measurements and estimates that lead to the final list of regions found: the base-pair level DNAm measured for each sample, the smooth functions used in the above model, and the regions where methylation differences are determined to be significantly different (Figure 1).

Note that these smooth functions are parameterized by bandwidth to adjust their smoothness, which in turn determines the size of DMRs found: e.g., large smoothing windows leading to longer DMRs detected. For data derived from Illumina HumanMethylation 450k beadarrays this methodology is implemented in the minfi [2] Bioconductor package.

Once these regions are determined, they would be exported, as BED file for example, and visualized on a genome browser to integrate with other annotations (for example, the location of CpG islands and genes), providing context and interpretation based on the relationship between estimated DMRs and other genome annotations. Unfortunately, at this point most information pertinent to the statistical analysis driving these inferences is lost (for example, the choice of smoothing parameters that determine the width of DMRs and therefore their overlap with other genomic features). Furthermore, interaction in the genome browser is not informed by statistical properties of the inferred regions (for example, navigating through regions in order of statistical significance).

Current tools make it difficult to simultaneously perform *statistically informed *visualization by allowing exploratory analysis of measurements and estimates across this multi-stage statistical procedure and *visualization informed *statistical analysis where parameters of this multi-stage procedure are explored based on integrative visualization.

Figure 1 shows how the design of Epiviz is based on supporting this type of workflow. Analysts can simultaneously visualize data across multiple stages of statistical analyses (at CpG level, the smooth functions in statistical model, and inferred DMRs) directly from data, through the Epivizr Bioconductor package. Changes in parameters of this statistical pipeline would be reflected immediately in the visualization. Analysts can also integrate annotation data (gene and CpG island location and expression data from the Gene Expression Barcode [4] project for multiple tissues). This paper describes in detail the design of our tool to support this type of analysis workflow.

Some of the ways Epiviz advanced the field of genomics interactive visual analysis include:

1. Introducing the first genomic data visualization software that brings code to interactive visualization by allowing computational environments such as R or Python to use it as an interactive display device and by allowing scripts stored externally (i.e. on source control providers) to be dynamically integrated into the framework. The Epivizr package is designed to integrate Epiviz with the state-of-the-art Bioconductor infrastructure for genomics statistical analysis.

2. Introducing the concept of community-contributed plugins for web applications through JavaScript dynamic extension. Epiviz is the first web-based visualization tool whose code base can be extended by actively incorporating third-party scripts. Combined with the *workspaces *feature, which allows users to persistently save, replicate and share analysis steps - including code customizations done in the UI - this opens the door to social collaboration within the genomic community, which has never been done before.

3. Creating the first genome browser that makes use of the following set of concepts and features simultaneously: brushing and linking, binning, supporting data transformations in the UI, predictive caching based on navigation, aggregating data from multiple sources - both cloud-based and local - and persistently saving and sharing data analysis steps as *workspaces*.

### Contributions

In this paper, we present specific extensions and features introduced posterior to the initial launch of our project and consequent publication, as well as aspects of the architecture that have not been described before.

1. Visualization code customization in the UI. Previous versions of Epiviz allowed users to create custom visualizations and data providers, as well as to override existing settings by storing scripts on the cloud and referencing them within the tool. A lot of data analysis workflows however, require customizations that are so simple, that creating a whole new visualization for them would be too much overhead. For cases such as these, Epiviz provides a window straight into the parts of the visualizations code that matter for the user, who can customize this code directly in the user interface, using a JavaScript code editor dialog.

2. Data transformations directly in the UI. In Epiviz, data transformations can be easily applied by coupling a computational environment to the interactive visualization environment. Also, new measurements can be created as combinations of existing ones using the *computed measurements *feature. However, in our work we recognize the need of being able to make such transformations as close to the user interface as possible, in order to optimize the data analysis workflow. For this reason, we expanded our framework's API to include a series of customizable and extensible transformations over data within visualizations. Measurement and feature sorting, aggregation, as well as dynamic coloring are a few examples of transformations currently available within our tool. Epiviz exposes a fully-featured JavaScript code dialog which scientists can use to define complex ways in which these transformations can be applied.

3. The extension of user *workspaces *to include user-defined code customizations. Reproducibility is an essential aspect of genomics data analysis workflows, which was one of the central concepts in the Epiviz design from the beginning. To keep our framework in tune with this concept, we extended the *workspaces *feature to include code customizations, for both visualizations as well as data transformations described above.

4. Securing third-party code. The introduction of the data and visualization code transformations described earlier, as well as storing these user-scripts into the database as part of workspaces poses security risks for server-side data integrity, as well as user information. For this reason, we implemented a series of security features, of which the most important is utilizing a code sanitizing library that ensures no malicious code will be executed within either the user session or our servers, and no such code will be stored on our databases.

5. A series of new visualizations, as well as advanced features of the existing visualizations, such as heatmap clustering, which we exemplify, alongside the new code features, in the *Results and discussion *section. The section also includes results derived from these new features and visualizations, which constitute important insights over the relationship between different genomic measurements.

6. We discuss the current challenges brought by data specific to genomic analysis and present an abstract data format designed with these challenges in mind. We describe ways in which the architecture of our framework takes these into account in defining a uniform data format We provide an in-depth description of the data abstraction and standardization of Epiviz, which is the cornerstone of our plugin API, facilitating the usage of the same visualizations for different data types, as well as using different visualizations for representing different dimensions of the same data type. The uniform data format is also an essential aspect of integrating data from different sources, both computational environments and cloud-based databases.

### Considerations about genomic data

Data used by bioinformaticians in data analysis is complex in its variety, heterogeneity, and size. Sometimes, this data consists of records mapped to a genome - for example, DNA methylation, or read coverage, which are commonly displayed in genome browsers [5-8]. In other cases, the main coordinate is replaced by time or some other user-defined dimension.

The size of genomics data also tends to vary greatly. Usually, a single dataset corresponding to the genomic data for a single sample will range from a few hundred records to a few hundred million [9], all of which can easily fit into the memory of a modern day computer. However, in the workflow of a single data analysis task, a scientist may involve a large enough number of datasets for it not to be feasible to load all data in the memory of a workstation at once, or even store it locally. For this reason, there now exist several large databases aggregating and making available various types of genomic datasets for a multitude of use cases [4,10,11]. There have been large efforts geared to storing efficiently and providing large selections of these datasets to bioinformaticians [12]. Often these sit behind genome browsers, which offer, to some extent, help with the dissemination of subsets of these datasets. However, in the past, no tool has been able to interactively visualize data from a multitude of these different sources simultaneously, and at the same time facilitate dynamic statistical analysis of new datasets derived from user experiments, residing locally.

In our work we recognize the need to analyze, model, navigate over, correlate and interactively visualize data that is both varied in terms of format and semantics, as well as source and size. Current data analysis workflows of individual researchers as well as research groups usually involve the usage of a multitude of tools as part of the same process, including a number of genome browsers and statistical environments such as R/Bioconductor or Python. To reduce the time spent by users switching between tools in the course of one data analysis process, genome browsers take one of two paths. The first is to duplicate data already available in other databases [13-15]; this approach is unfeasible when taking into account the rate at which new data becomes available [11,16], the sizes of some such databases [4,17], as well as the efforts aimed at making these databases efficient in terms of query time and data format [12]. The other path is to serve data directly from the cloud [6]. We find the latter preferable, given the data considerations made earlier. But, in addition to genome browsers, data analysts often revert to computational platforms, for quick access to data widely used in their scientific community as well as quick manipulation and complex modelling of small datasets. For genomics data, the R/Bioconductor framework is a state-of-the-art platform for implementation and dissemination of computational and statistical analysis methods.

However, before Epiviz, no existing tool by itself was able to accomplish both of the following: 1) integrate simultaneously data from different genome browsers without replicating it on the local database, and 2) bridge the gap between genome browsers and computational environments, by coupling interactive visualization with statistical data modelling. Genome browsers sit in front of large databases and are highly interactive, but have little to no data transformation capability. Computational environments on the other hand, are powerful data modelling tools, but do not expose interactive visualizations, nor are they capable of easily manipulating extremely large datasets, being limited to the physical memory of the local machine.

One of the purposes of Epiviz is to make different datasets in various locations and formats easy to access and manipulate in the same tool. Through Epiviz, we propose a system that is able to visualize datasets directly from their existing locations, as well as custom user data residing on the local machine, side by side. Not only this, but the users are now able to model and change their custom data in a computing environment of their choice and immediately visually explore these changes, all in the same view, without the need to switch between tools or to constantly upload new versions of transformed data to a web server. Epiviz introduces a framework that features a data provider API which can be used to integrate both data available through online services, as well as data loaded in the memory of a computing environment. In addition, users of Epiviz gain the benefits of new data sources and formats available only to services within these environments [18-20]. These services provide uniform access to a large number of data sources to support data integration by providing infrastructure that supports a large variety of data types based on community standards (Sup. Table 2 in Additional file 1). Development of interactive and integrative visualization tools that are able to interact with frameworks such as R/Bioconductor is a strong motivation for Epiviz. Through them, it is assured of high visibility and impact in the scientific community. Tools that directly benefit from our system include a number of frequently used, state-of-the-art methods for a) ChIPseq (DiffBind [21]), where iterative visualization of data and results of peak-calling algorithms is necessary; b) RNAseq analyses using DESeq [22], edgeR [23], or limma [24], and Cufflinks/Cuffdiff [25] through the cummeRbund package, where both location-based coverage and feature-based expression levels are required; c) methylation analyses using BSmooth [3] or minfi [2], where location-based analysis at multiple genomic scales is important.

## Implementation

### Bringing code to interactive visualizations

Coupling between computational environments and visualizations has been done before, to some extent, either in areas of computer science unrelated to genomics, or for different audiences than the one targeted by Epiviz. In this section we briefly describe two categories of tools that attempt to do this, underlining aspects that we considered worth replicating for our purposes, and ideas we decided to take a step further. Combined with the interactive features of a genome browser, these make Epiviz the first tool of its kind.

#### Web-based computational environments

One category of tools that has gained a lot of traction in the past years is represented by web-based computational environments, such as IPython Notebook [26] and RCloud [27]. They are much similar to regular computational environments, but in addition, they combine code execution in browser, plots, and rich media. What we find particularly useful about these tools is the ability of users to create custom notebooks, and share them with each other. This is done either via individual files that can be viewed using an online notebook viewer, or using cloud-based source control providers such as GitHub. This collaborative approach has been proven extremely effective for the audiences of these tools, consisting mainly of programmers and data analysts.

But neither of these tools can substitute a genome browser, because they are not specialized on interactive visual exploration of genomic datasets. They feature a set of static charts, the same as regular computational environments, but operations specific to genome browsers, such as navigation, or brushing, to link data across different charts are not naturally supported.

#### Shiny

Shiny [28] is an interactive web application framework for R, which features a set of predefined web widgets which can be used to visualize custom sets of data loaded in memory. What is especially useful about this tool is a feature called *reactivity*, which consists of binding web controls to R functionality that responds to user actions on the web interface. This opens the door for R to visualizations that are more interactive than conventional static plots. This, along with the fact that its native environment is R, makes the tool extremely popular within the computational genomics community.

Although it is a great addition to the R computing environment, Shiny is a general framework that cannot replace genome browsers in a data analysis workflow without significant extensions. It is not built to link data across charts using brushing, nor does it support navigation over subsets of the same data. In addition, custom visualizations and visual optimizations cannot be built directly in R - knowledge of JavaScript and visual libraries built on top of it is required to create advanced complex views. In addition, Shiny is not built to display partitions of data at a time, but rather, relies on the entire data to be loaded in memory of R. Also, there is no easy way to put together subsets of data from a multitude of large databases.

#### Visualization systems with similar functionality

Harger et al. [29] and Zhang et al. [30] provide two comprehensive surveys and comparisons of the state-of-the-art visual analytics open-source toolkits and commercial systems respectively taking into account a wide variety of features, from visualizations offered, to analysis functionality and portability. Based on these studies, the Titan [31] toolkit and the system Spotfire [32] stand out through their extensive combination of *visualization *and *statistical analysis *functionality. All other tools analyzed in these surveys lack support for *multivariate statistical analysis *- the statistical analysis of data in three or more dimensions (for example, *dimensionality reduction, clustering*, etc.), which is essential for genomic data analysis workflows.

#### Titan

Titan is not in itself a visualization tool, but rather a toolkit providing an environment for data visualization and analysis. Unlike most other desktop-based visualization toolkits, it is OS-flexible, offers a good set of multiple coordinated interactive visualizations, as well as a computational facet for C++, Python and TCL. The architecture is also extensible by allowing users to create custom plugins for data transformations.

In contrast, one of the goals of our efforts in developing Epiviz was to provide support for frequently used state-of-the-art methods for genomic data analysis. This is currently best addressed by R/Bioconductor, through an extensive suite of libraries and packages that are able to both manipulate different types of genomic data (Sup. Table 2 in Additional file 1), and facilitate workflows for a number of established domain-specific analysis methods, as outlined in *Considerations about genomic data*. The Titan toolkit is bound to C++, offering limited ways in which a connection to the R/Bioconductor infrastructure could be established. In addition, customizing the code of Epiviz is an easy task partially because of our choice of JavaScript as the main programming environment for the framework. With Titan, in order to create a plugin, one needs to instantiate and build the entire framework on the local machine. In contrast, the current design of Epiviz requires no download, being able to interpret and execute third-party code from cloud-based locations such as GitHub. Our option also implicitly solves a security concern since JavaScript is given limited access to the user file system, which is ensured by web browser vendors. Desktop-based software on the other hand, is vulnerable to a wide variety of security vulnerabilities.

#### Spotfire

Spotfire is a commercial desktop-based data analysis and visualization tool which also comes with a wide variety of analytic features, like brushing and linking, multivariate statistical functions, as well as a feature which allows users to define custom data transformations by writing IronPython code directly into the UI. As powerful as it is, the tool comes with a number of limitations which make it difficult to use for genomic data analysis workflows. The most evident is the fact that the tool is OS-dependent, being able to run solely on Windows. This is a serious drawback for the genomics community, where more than half of the users work on either Macintosh or Linux (Sup. Figure 3 in Additional file 1). Like Titan, Spotfire does not natively support common genomic data types, and relies on loading the entire data in memory, making it unfeasible to use for extremely large datasets.

#### Epiviz

Epiviz brings code to visualizations in ways similar to those described in the previous sections.

First of all, it features a WebSockets API similar to that used by Shiny, which allows communication between the web framework and any environment that implements the corresponding endpoint protocol. This API is used by Epivizr, a Bioconductor package that permits communication between the R computational environment and the Epiviz user interface.

Secondly, Epiviz exposes a plugin API for both data providers and visualizations, so that new data sources can be easily added as needed, and new visualizations can be defined to display the same data from different perspectives. Custom JavaScript code for new visualizations or data providers, can be plugged in on-the-fly using GitHub Gists, similar to the way IPython and RCloud incorporate custom user notebooks.

Finally, a set of code features provides a gateway into the parts of JavaScript code that matters for the effective transformation of visualizations and data depicted in them. These are described in detail in *Visualization customization in the UI *and *Data transformation in the UI*.

### Epivizr

Epivizr is an R/Bioconductor package that uses the data provider WebSocket protocol to connect to the Epiviz framework. Through it, Epiviz makes requests to the R environment so data in R objects is served in response. All data sources catalogued by the AnnotationHub Bioconductor resource are available for integration as measurements via Epivizr: the UCSC genome browser database [10], Ensembl [11], and BioMart [19]. Infrastructure from the core Bioconductor team and hundreds of contributed packages are used in a large number of projects analyzing data that ranges from expression microarrays to next-generation sequencing. Due to Epivizr, users of Epiviz immediately benefit from the fundamental data structures exposed by Bioconductor in their analyses. Conversely, developers of new methods in R and Bioconductor have now access to an interactive way of visualizing data at each step of development.

Epivizr features updating, filtering and subsetting operations on R objects that trigger updates in their corresponding visualizations in Epiviz. One of its most important capabilities is that it supports interactive exploratory browsing by, for example, allowing users to navigate in order through a set of genomic regions defined in R, using the *slideshow *feature. Thereby, users can rank regions of interest according to some predefined, or computed attribute. A canonical example is navigation through regions of differentially expressed genes from an RNAseq experiment obtained from packages like DESeq or EdgeR.

### Software extension using JavaScript dynamic code interpretation

One powerful feature of JavaScript is its ability to evaluate strings of text into runnable code, which Epiviz makes use of, to dynamically incorporate custom user logic into the framework. In this section we expand over the different kind of functionalities based on this feature available in Epiviz. Also, in the *Results and discussion *section we discuss the security implications of taking this path, as well as our approaches to addressing them.

#### External scripts

One way in which we make use of this capability is by providing an extension in the API which permits automatically incorporating user specified external scripts that can override existing functionality, such as visualizations, data providers and settings, or define new ones. The new visualizations, data providers and custom settings are ready to use immediately, alongside predefined ones. On launch, Epiviz first loads the base framework logic and searches for scripts specified using user-provided parameters. Epiviz then executes the code in these scripts in sandbox mode and UI elements are immediately updated. Epiviz supports the GitHub Gist API, which makes it possible for users to specify code stored on this source control provider. Using this functionality, users can collaborate on a set of scripts simultaneously, sharing their work while using a common set of workspaces as the functionality contained in the scripts evolves.

**Figure 2:**
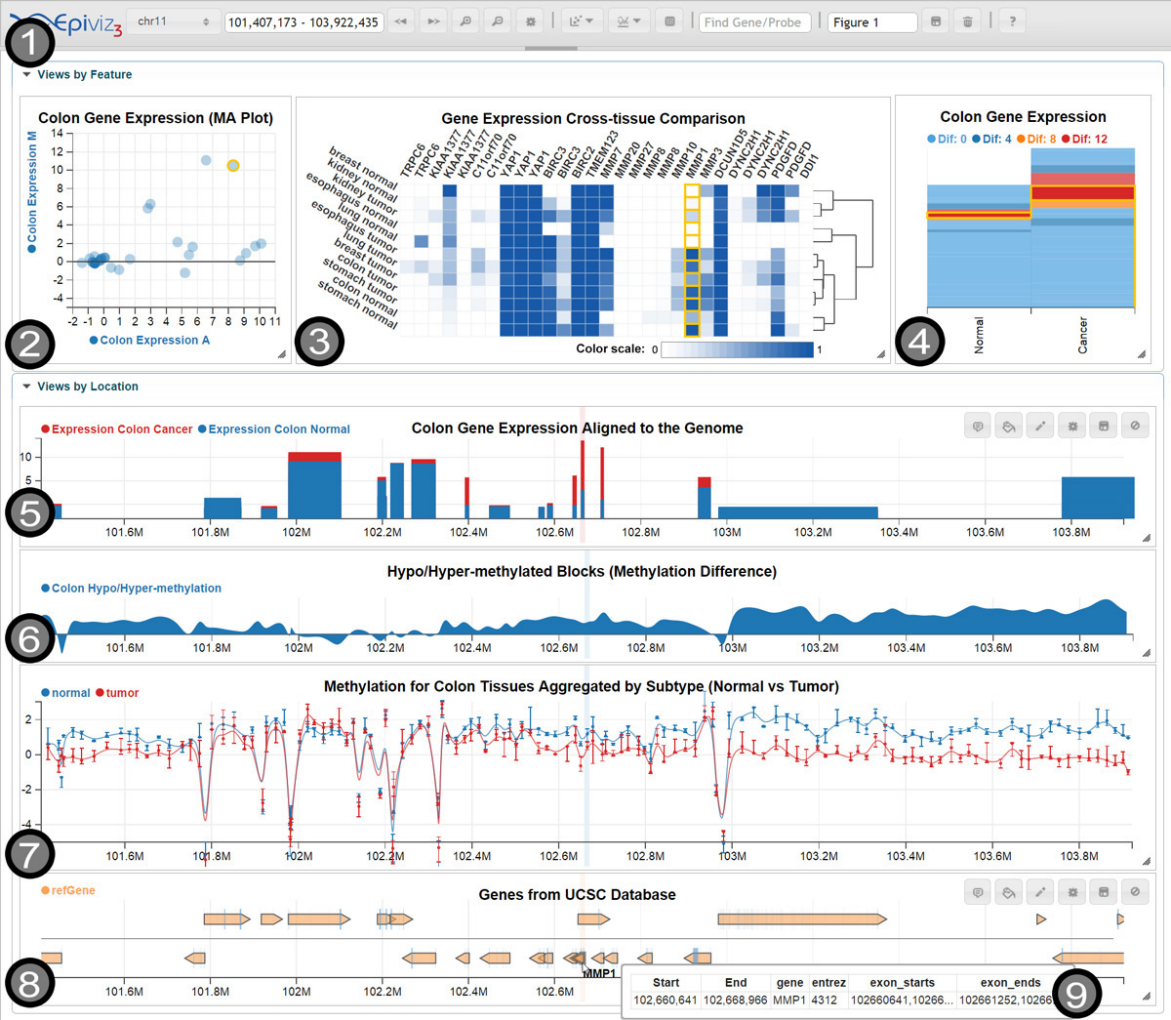
**Screenshot of Epiviz**. It shows the main elements available within the tool: 1) the *main toolbar*, featuring all UI controls; 2) a *scatter plot*, showing two *computed measurements*: the average and difference between colon gene expression for normal and cancer tissues; the code for this plot is customized in the UI to show a line at *y=*0, that separates genes with positive and negative differences; 3) a *heatmap*, showing values from the Gene Expression Barcode [4] comparing the normal and cancer expressions for different tissues. Using its clustering feature, we notice that tumors tend to group separately from normal tissues; in addition, the clustering result seems to be determined by a small number of genes, namely MMP1, MMP3 and MMP10; 4) a stacked plot, showing two columns for normal and cancer gene expression; it uses the *color by *transformation, to highlight genes with various expression differences. This plot offers several insights: first, that overall expression tends to be higher for cancer than normal tissues; second, it allows us to immediately spot the differentially expressed genes, by brushing over the blocks colored in *deep red*, corresponding to them; 5) a custom track defined in a plugin hosted on GitHub Gist, showing blocks aligned to the genome, with height corresponding to the expression of the genes; 6) a *stacked track*, showing a *computed measurement*, corresponding to the difference between normal and cancer methylation; this track offers an insight over the hypo/hyper-methylated blocks; 7) a *lines track*, showing DNA methylation for normal and cancer colon tissues; the track uses the *group by *transformation to aggregate three normal samples and three tumor samples, and displays error bars to show the variation of methylation for each group at each data point; in addition, it uses *basis *interpolation to smoothly connect the available data points; 8) a *genes track*, showing human genome genes fetched from the *UCSC database*[10] using a *data provider plugin *stored externally on GitHub Gist; 9) a tooltip showing details on demand for the gene MMP1. The highlighted items correspond to the brushing feature, triggered while hovering over the MMP1 gene in the genes track. The feature links all visualizations together by genomic location. http://epiviz.cbcb.umd.edu/?gist[]=160e8b84795603961b9f&gist[]=5a88f39caa801e58b8ae&ws=GJU2bfURaUd

Visualization plugins can be easily created using the Epiviz Visualization API, which exposes a series of interfaces and base classes. These classes implement basic functionality, like drawing axes, creating a main SVG canvas where drawing is done, drawing legends etc., which can in turn be used by plugins that only need to implement a *draw *method, greatly simplifying the complexity of plugin code. Figure 2 shows an example of an externally defined visualization that follows this API, and whose code is hosted using GitHub Gist.

New data providers can be specified in Epiviz using the same mechanism, and a Data Provider API, designed to allow users to dynamically plug in custom sources of data. Using the data provider plugin mechanism, Epiviz can display data located remotely or on the local machine. Conceptually, Epiviz data providers represent proxies to real data sources. For example, the WebSocket data provider enables connections with R/Bioconductor through the Epivizr package. Different data providing services are interfaced through an API that de-centralizes data storage by allowing users to easily integrate external data sources. Figure 2 contains a track that displays human genome genes from the *refGenes *SQL table, in the UCSC database. This is done through a custom data provider, plugged into Epiviz using the *gist *feature.

#### Visualization customization in the UI

Epiviz also introduces a mechanism that permits users to customize charts' code directly in the UI, as well as to define simple data transformations. Using this, users can alter individual visualizations in place to match their needs. The code of chart *instances *can be modified such as to incorporate additional functionality per user needs. For example, the scatter plot in Figure 2 contains a line at *y*=0, separating positive and negative gene expression differences. In Sup. Figure 4 in Additional file 1 we show the dialog where users can edit the visualization code, and the code necessary to apply this particular transformation to the scatter plot.

#### Data transformation in the UI

The same type of functionality is used to apply data transformations for individual visualizations. These define ways in which the data that comes into a visualization should be transformed prior to rendering. Currently, Epiviz provides support for the following transformations: 1) *filter *by data object properties; 2) *color by *measurement or coordinate properties; 3) *group by *measurement properties; 4) *order by *measurement properties.

**Figure 3:**
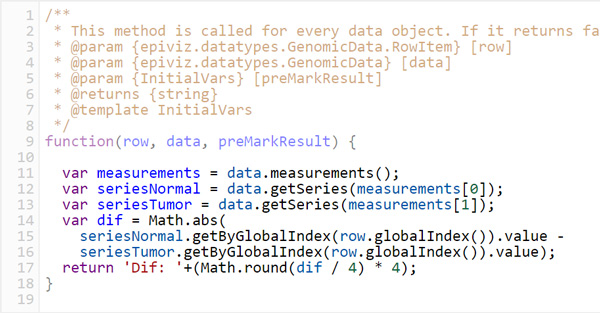
**Custom *color by *transformation for the stacked plot**. This code computes the absolute difference between the two measurements - for example gene expression normal and cancer - in the plot, and splits it in increments of 4. The resulting plot will colour genes with different colours, each corresponding to its expression difference.

In the following lines we expand on these transformations. Each of them exposes two functions that users can implement using the code editor controls in the *code dialog *(Figure 3, Sup. Figure 4 in Additional file 1). The first is called before any transformation, and is used to define and initialize variables to be used throughout the transformation; the second function corresponds to the actual transformation, and is called for each object, measurement, or feature coordinate in the selected genomic region, depending on the transformation.

In the *filter by *transformation, the inputs correspond to records in a *data source *(Sup. Figure 1 in Additional file 1). Each input contains coordinate information, as well as a feature value. The *filter by *function yields a *Boolean *with the following semantics: 1) a returned value of *true *signifies that the item should be drawn in the visualization, while 2) *false *means that it should be hidden.

The *color by, group by *and *order by *transformations, all have the same signature (Figure 3): they can be set up to take as inputs either *data source *records, *measurements*, or *feature coordinates*. Based on the input, the function returns a *label*, in the form of a text string or a number. Labels are used by each transformation accordingly. For example, *color by *will use them to color the objects with the same label with the same color. *Group by *uses a user-selected aggregation function to aggregate all objects with the same label into one visualization object. Finally, *order by *sorts objects in the visualization according to the lexicographic order of their corresponding labels.

For example, the stacked plot in Figure 2 uses a *color by *transformation, which is used to highlight genes with various expression differences - *0-4*, light blue, *4-8*, dark blue, *8-12*, orange, and *>12*, red. The code necessary to apply this transformation is depicted in Figure 3 which shows a screenshot of the contents of a code transformation dialog.

Another example is the *lines track *depicted in Figure 2 which uses a *group by *transformation, where the two labels are *tumor*, if the measurement corresponds to a tumor sample, and *normal *otherwise.

The Epiviz API also implements a simple computing language for creation of new measurements from combining existing ones. We call the result *computed measurements*. These differ from the previously mentioned transformations in that they act like regular measurements in the framework, and are available globally for all visualizations to use. For example, based on the gene expression values for normal and cancer colon tissues, we generated a particular kind of scatter plot also known in genomic data analysis as an *MA plot *- the *x *axis shows the average gene expression for normal and cancer colon tissues, while *y *corresponds to the difference. This plot is used in the use case presented in the *Results and discussion *section and shown in Figure 2. Dudoit et al. [33] offers an in-depth analysis of the motivation behind choosing this kind of plot for differential gene expression analysis. Computed measurements values are calculated lazily, as needed by visualizations, just for data represented on the screen.

In the use case illustrated in the *Results and discussion *section, we provide several examples of each of these features - chart code customization, data transformations, as well as computed measurements.

### Visualization system concepts put together for the first time in genomics

Apart from bringing code to visualizations on various levels, Epiviz also uses a series of design choices and features of which some have been used before for genome browsing, others for other various types of systems. What makes Epiviz stand out is that it is the first software to put all of them together in an integrative genomics interactive visual software. In this section we present some of the most important of these choices, underlining, where necessary, the motivation that led to their development, as well as benefits that follow their implementation.

#### Visual encodings

The Epiviz UI offers data scientists a combination of *multiple coordinated views *and *overlays*, featuring *brushing *and *linking*. The main goal behind this is to enable both data comparison and visual validation in order to help users extract insights and gain both an overall and detailed understanding of the data. Epiviz provides out-of-the-box visualizations that are both *feature- *and *genomic location-oriented*, to help provide a multidimensional comprehension of the explored domain. Both types of visualizations offer in turn different kinds of graphical representations; for example some of the available feature-oriented views are the *heatmap *and *stacked plot*, while some of the available location-oriented views are the *genes *and *line tracks *(Figure 2). This differentiates Epiviz from most other genome browsers, which usually feature only *genomic location-oriented *visualizations.

All graphics are rendered using Scalable Vector Graphics (SVG), an XML vector format that all modern browsers can interpret. Choosing this format allows users to treat objects in charts independently, as direct representations of data [34], as well as to perform specific operations on them, by customizing their properties - shape, color, size, stroke, transparency, etc. This opens the door to a wide variety of options available directly to visualizations, of which perhaps the most important are *brushing and linking, object tooltips*, and the ability to save views as both vector and raster static images.

#### Brushing

Through brushing, users have the ability to visually link data from all visualizations on the screen (Figure 2). By hovering over/selecting a particular object in one chart, related objects are automatically highlighted in all other charts as well. The unified data types used in Epiviz include identifiers for *data source groups *which declare keys for each set of observations, to establish data relationships used in the brushing feature. Therefore, all *data sources *from the same group are assumed to have the same keys. In the absence of keys, we use feature coordinate overlap to establish these relationships as well. Notice that this design is extremely flexible since keys defining data relationships are defined dynamically. Brushing is available in Epiviz due to the choice of SVG as the rendering mechanism, since each object on the screen corresponds to an HTML element in the DOM. Hovering or clicking on an object thus triggers events that all visualizations listen to in order to decide which objects will be highlighted at their end.

#### Collaboration through sharing of analysis steps

One important functionality essential to scientific data analysis, and yet inexistent in current genome browsers, is that of persistently saving and sharing steps of an analysis within the scientific community. Often times, operations that should be straightforward, such as replicating results presented in publications pose big challenges and come with heavy overhead. Through Epiviz, we take the first steps in the direction of simplifying this process, through a feature called *workspaces*. Workspaces were first introduced in our previous work [1]; however, following that, we extended them with new analysis state components, such as code customizations and data transformations, so that any user action within the software could be tracked and reproduced.

Workspaces contain complete information needed to reproduce analysis states in Epiviz. All visualizations on the screen, including their customizations (screen coordinates, code changes, data transformations and color palettes), and the current genomic location in view are included as part of a workspace. Workspaces are stored in a database on the Epiviz server hosted at the University of Maryland. To save analysis steps, users need to authenticate using an OpenID account. Once logged in, users can create new workspaces and replicate existing ones shared with them. Storage management for persistent workspaces is part of the data provider API. Source code for this type of server is publicly available on the Epiviz project page and can be installed on a standard PHP/MySQL system to provide the same functionality if users desire to keep their workspaces private on a local server. Once created, a workspace is associated with a unique id that can be used to share individual work with other users, through permanent hyperlinks. Using the workspace id, any user can view a particular workspace, and copy its contents to their own account. This mechanism can be used for either sharing data analyses between users or even for referencing figures in publications (Figure 2).

## Results and discussion

### Exploring the relationship between gene expression and DNA methylation

We present a use case that highlights the most powerful features of Epiviz, with an emphasis on the ones introduced following the work presented in Chelaru et al. [1]. We used Epiviz to explore the relationship between DNA methylation and gene expression within normal and tumor colon tissues. Our goal was to examine regions in the genome where the difference in gene expression between the two is large.

**Figure 4:**
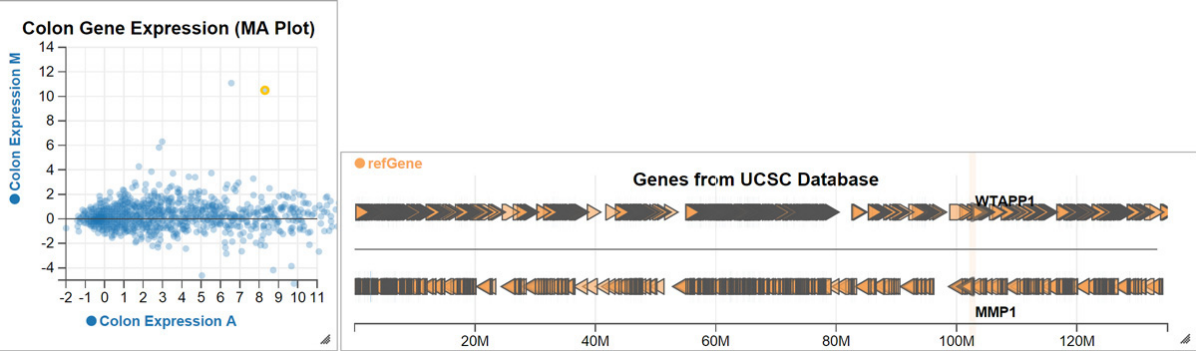
**An overview of gene expression in chromosome 11**. The *scatter plot *shows colon normal and tumour gene expression average on the *x *axis, and difference on the *y *axis. The *genes track *shows genes fetched from the UCSC database, using a *data provider *plugin hosted on GitHub Gist (http://gist.github.com/5a88f39caa801e58b8ae). The highlighted data point in the scatter plot corresponds to a gene expression difference outlier. Using the *brushing *feature of Epiviz, we link this outlier to its corresponding gene in the *genes track*. http://epiviz.cbcb.umd.edu/?gist[]=5a88f39caa801e58b8ae&ws=gdmUH1ANl3m

We started with a *genes track *showing gene models from the UCSC database. To fetch this data, we used a custom *data provider *plugin stored on GitHub Gist. In order to find regions in the genome where expression differences are large, we used two *computed measurements*, corresponding to the *average*, and *difference *gene expression, respectively, for RNA-seq data from chromosome 11. We displayed these using a *scatter plot*. In order to better observe expression differences, we dynamically customized the code of the scatter plot to show a line at *y *= 0 (Figure 2). We identified two genes with large expression difference by selecting the outliers in this plot from all genes in chromosome 11: *MMP1 *and *MMP3*. The brushing feature allowed us to observe that these outliers are also adjacent in the genome. To find the exact genomic locations of these genes, we hovered over them to trigger a tooltip (Figure 4, Figure 2).

We zoomed into a smaller genomic region to examine these genes at high resolution. To check whether expression differences are consistent across tissue types, we added a *heatmap *with aggregated expression data from the Gene Expression Barcode [4], for six different tissue types, both normal and tumor: colon, stomach, breast, kidney, lung and esophagus. We used this visualization's *clustering *feature to group tissues based on gene expression similarity within this genomic region. The high expression differences for the *MMP1 *and *MMP3 *genes, between normal and tumor tissues, across tissue types, played a decisive role in the clustering result (Figure 2, Sup. Figure 5 in Additional file 1).

Next, we added a new visualization called *stacked plot*, showing two columns, corresponding to the summed gene expression for normal and cancer tissues respectively. This visualization stacks values for different genes, one on top of the other, depicting each gene with a different color. Using a *color by *transformation, we customized this plot to color-code different genes according to the expression differences (Figure 3). Analyzing the result yields a couple of insights: first, that overall gene expression tends to be higher for cancer tissues; and second, that genes with high expression difference tend to be collocated in the same region of the genome (Figure 2).

To examine gene expression along the genomic coordinate in relation with DNA methylation, we created a custom track plugin, which we stored on GitHub Gist. The track displays genes as blocks aligned to the genome, whose height corresponds to the gene expression. We also added a new visualization called *stacked track*, which we used to display a *computed measurement*, corresponding to the difference in methylation levels between normal and cancer samples (Figure 2, Figure 3). The data for these measurements corresponds to base-pair-resolution smoothed methylation log ratio resulted from sequencing of bisulfite-converted DNA [35]. The advantage of this type of visualization over the traditional *blocks track *which we used in the previous version of our work is that this offers not only information about the location of *hypomethylated blocks*, but in addition, it provides a two-dimensional high resolution understanding of the structure of these blocks. For example, examining these two tracks side by side, we were able to extract a number of insights: first, the density of genes increases as methylation difference decreases; second, gene expression for both normal and tumor samples increases in regions where methylation difference is smaller; and third, we were able to differentiate between hypomethylated regions by their level of auto-correlation (smoothness) at base-pair level, which links well in this region with gene expression differences.

Finally, we created a line track showing the methylation levels for six different samples, three normal and three tumor. We used the *group by *transformation, to aggregate normal and cancer samples together respectively (Figure 2). A high resolution view over the data allowed us to further differentiate between the two hypomethylated blocks in view: the first, ranging from 102.3 Mbp to 102.9 Mbp, and the second, ranging from 103 Mbp onward.

We discovered that although at the block-level there is an overall difference between cancer and normal methylation, the degree of auto-correlation in the methylation data at base pair resolution varies within the block. Furthermore, we observed that genes that show high differences in expression tend to collocate with regions of low methylation auto-correlation (lower smoothness) while genes that are not differentially expressed collocate with regions of high methylation auto-correlation (high smoothness). This analysis suggests that understanding the relationship between expression and methylation within long epigenetic domains requires that methylation data is analyzed at multiple genomic resolutions. Using Epiviz, it was easy to switch between low and high resolutions to facilitate these type of multi-resolution analyses.

### Notes about software security

One challenge that comes with allowing Epiviz to incorporate and run third-party scripts consists of the security risks associated with SQL injection and cross-site scripting (XSS) [36]. The main concern is that, having access to private user information, third-party scripts could be used to compromise user content, privacy, and sensitive data. In this section we underline the ways in which Epiviz addresses these concerns in order to provide a safe data analysis environment.

Epiviz features a server side component, and a client JavaScript component, the latter containing the entire framework functionality, described in this paper. The server side component contains a web service for a number of public epigenetic datasets, similar to those hosted on other genome browser servers, as well as private Epiviz user information, such as their OpenID account data provided when signing up, and workspaces associated to it. The public sets of epigenetic data are served in a read-only fashion, no changes to it being permitted to users accessing the service. OpenID information cannot be retrieved using the web services endpoint; in addition, information about a particular user cannot be modified externally after the user has logged in for the first time, having successfully authenticated using the corresponding OpenID provider. The only information that can be both retrieved and modified externally on the server side is that of user workspaces. Changes to workspaces can be made within the session of their owner. All database access for the webserver is achieved through PHP prepared statements [37] which are guaranteed safe from SQL injection.

This implementation leaves room for only one type of potential attacks. Malicious external JavaScript code, being incorporated into Epiviz using the dynamic code interpretation feature, might gain access to the user session and potentially compromise workspaces information, the only kind of private information that can be accessed and modified externally. Alternatively, it could extract private information such as that stored in user cookies, and transmit it to a phishing server using, for example, AJAX calls. This constitutes a great vulnerability, and therefore, we needed to find a solution which would allow the execution of third-party scripts in a sandbox mode, with no access to sensitive information or actions that might compromise data integrity.

To address this vulnerability, we used a JavaScript sanitizing library called Caja [38], which allows third-party scripts to be executed by the same JavaScript runtime environment as the framework code, but in sandbox mode, with restrictions defined within the host script.

Whether the scripts are stored externally, or they consist of custom code inserted using the web-browser code editor, they are executed in protected mode, which effectively defends against XSS attacks. This security feature allows Epiviz to be the first visualization tool to allow its users the wide range of power that comes with expanding functionality through third-party plug-ins and code customization.

### Future research directions

#### Performance and optimization

There are three ways of addressing the limitations brought by the choice of JavaScript as the main programming environment for our framework. In this section we describe some which we are looking to implement in our future work: 1) a clearer separation of data processing operations from visualizations, to better use with web workers; 2) the use of the WebGL technology, which comes with all the power of gaining direct access to the GPU [39]; 3) as the new technology WebCL [40] becomes available, and execution threads are introduced into the language, move some of the most CPU-consuming operations of Epiviz into secondary execution threads.

The choice of SVG for rendering visualizations also comes with a number of limitations which we describe in the supplementary material. In Chelaru et al. [1] we discussed a number of optimizations for individual charts, as well as their effects over render latencies. What is not said there is that these optimizations cannot, because of the design decisions of Epiviz, be generalized so that new visualizations can take advantage of them. A solution for this drawback, which we are looking at implementing in future versions of our software, is to alternate between raster views and vector views, depending on the amount of data loaded in memory. We anticipate this approach to significantly improve user experience for the visualization of extremely large datasets.

#### Research directions in collaboration

Epiviz has taken a few preliminary steps in the direction of providing a collaboration-friendly environment. Two features in particular are aimed to help teams that work on a joint data analysis project: 1) workspaces, which provide a way of storing analysis steps persistently, and sharing them among the community; 2) an API that has support for custom software plugins created within the scientific community, stored on GitHub Gist, which can be used to share visualizations and data providers among users. In addition, as the entire code base of Epiviz is open-source, users from the scientific community are able to report errors or new ideas, as well as contribute to the development of the project. However, these represent only our preliminary efforts in a long-term plan of creating a truly collaborative data analysis environment and by no means do we consider them complete. In this section we briefly introduce the next two steps we mean to take in this direction, based on previous research on this topic [41] as well as related examples [26,27] where approaches have proved to provide a high level of collaboration, enhancing usability, transparency and interaction among users.

A first step is to centralize workspaces, as well as custom user code used for both plugins and data transformations, in a webpage similar to those provided by IPython and RCloud for user notebooks. We intend to allow our users to browse existing workspaces and plugins, copy and extend them, as well as rank them using a starring system. The goal for this feature is to introduce transparency and awareness within the Epiviz community, allowing users to dynamically interact with each other and expand the usability of the framework.

The second step is to introduce *same-workspace concurrent collaboration*. The current functionality already allows the same Epiviz instance to pull data from multiple sources, of which several can be either R or Python sessions. These sessions can be located on any network-accessible machine. This opens the door to a peer-to-peer type of collaboration similar to that of Google Docs, where multiple users can connect to the same data sources and computational environment sessions, and interact from different workstations over the same joint workspace simultaneously. Changes made to the workspace by one user will be immediately propagated and become visible to all other users connected to the same R session. The current architecture of Epivizr is one step away from permitting this kind of functionality. The only thing missing is an awareness component within the Epivizr package which will track the activity of each connected user, and propagate it to all the others. The peer-to-peer approach also implies that no centralized server would be necessary for this architecture, delegating that functionality to the R/Epivizr sessions instead.

#### Incorporating new technologies

Epiviz was designed with the goal of easily incorporating new technologies, both on the data integration side (as new infrastructure in Bioconductor is designed to incorporate new genomic data types) and the visualization side. For example, new technologies such as *htmlwidgets *[42], released after the initial launch of Epiviz, consists of an R package that provides a framework for creating R bindings to JavaScript libraries. Users and developers are tasked with creating wrappers around JavaScript libraries, in order to use them directly in R applications. The package can be used alongside Shiny to facilitate two-way communication between R and JavaScript. The design of Epiviz permits the creation of bindings for the Epiviz *visualizations tier* using *htmlwidgets*, to facilitate the use of our visualizations independently of genomics data analyses. Generic visualizations included in Epiviz, such as the heatmap or scatter plot, which have grown to be very feature-rich - exposing options for coloring by data features, clustering, binning, brushing and linking, etc. - can thus be made available to the entire R community in a lightweight fashion, without the tight coupling to the entire Epiviz framework, or the Epivizr Bioconductor package.

## Conclusions

We gave an overview of the motivations that led to the development of Epiviz as well as a series of design decisions and features that have never been put together before genomics interactive visual analysis. Epiviz is the first genomic data analysis software that brings code to interactive visualization, bridging the gap between computational environments and genome browsers. The software also sets a precedent for genomic data analysis collaborative workflows by enabling reproducible and shareable steps, and allowing custom user code to be dynamically incorporated, while guaranteeing the security and integrity of user data.

## Availability and requirements

Epiviz is available at http://epiviz.cbcb.umd.edu. Epivizr is available as a Bioconductor package (http://bioconductor.org). Documentation is available at http://epiviz.cbcb.umd.edu/help.

## List of abbreviations

DNAm: DNA methylation; DMR: differentially methylated region.

## Competing interests

The authors declare that they have no competing interests.

## Authors' contributions

FC and HCB designed the software, analyzed data and wrote the manuscript.

## Supplementary Material

Additional File 1 Click here for file
