# Supplementary material for: Epiviz: a view inside the design of an integrated visual analysis software for genomics
Source: BMC Bioinformatics. 2015 Aug 13;16(Suppl 11):S4. doi: 10.1186/1471-2105-16-S11-S4 (PMC4559604; doi:10.1186/1471-2105-16-S11-S4)
Supplement: Additional File 1 [file 1471-2105-16-S11-S4-S1.pdf]

Florin Chelaru and Héctor Corrada Bravo

Supplementary material

Contents

Other visualization tools for genomics ... 1
Data abstraction and standardization ... 1
Overview of performance optimizations ... 2
Performance limitations ... 2
Software security through code sanitization ... 4
References ... 4
Supplementary figures ... 6
Supplementary tables ... 8

Other visualization tools for genomics

A number of existing tools approach some of the capabilities targeted by Epiviz, but fall short. For example, the Human Epigenome Browser[1] provides powerful visualization and data integration capability, but is not directly integrated with a computational environment. Epiviz, can not only communicate with computational frameworks like R and Python, but the AnnotationHub resource accessible through Bioconductor also features integration of curated data sources in a uniform manner supporting interactive exploratory workflows not offered by the Human Epigenome Browser. Galaxy[2–4] is another tool that provides excellent support for pipeline workflows, but providing a limited set of visualization options. The design of Epiviz facilitates the creation of user-defined visualizations, which can immediately be used alongside the existing ones. Also, the code of existing visualizations can be customized directly in the UI. A related tool, cBio Portal[5] allows querying and visualizing data in a uniform manner, but is targeted just towards cancer genomics. In contrast, Epiviz provides an extensible framework which works for generalized workflows.

Current tools for the analysis of large genomics datasets usually target one of two audiences: either programmers and data analysts, who interact with data mostly through code and scripting, or biomedical scientists, who usually interact with data through graphical user interfaces. Conversely, there has been a significant push from educational and scientific institutions, including the NIH, to increase the computational literacy of biomedical scientists in preparation for work in a data-intensive field like high-throughput genomics[6, 7]. By continuing to target graphical user interfaces as the *only* way in which biomedical scientists can interact with data, current tools are not in tune with this trend. As a core design component of Epiviz, we added *code* as an exploration interface to genomics data at various different levels which we describe in detail in this paper.

Data abstraction and standardization

One of the most important design decisions in Epiviz was to introduce a series of data structures built on top of an open-ended standardized data format, used to represent a most common known genomic data types. The necessity for this design decision stems from the heterogeneity of genomic data types and the rate at which new ones are released. The unique data format permits different modules of Epiviz to interact with one another without predefining a protocol specific to just those modules. This simplifies the creation of new components, plugging them into Epiviz, and extending existing ones. The standardized data format is the key to our tool's customizability and extensibility, yielding benefits at four different levels: a) allows Epiviz to integrate and aggregate data from different sources, b) allows representing different measurements into the same visualization, for comparison analysis, c) allows representing the same measurement in different visualizations simultaneously in order to explore different aspects of its features, and d) allows Epiviz to expose an API that defines a plug-and-play fully featured chart interface, that users can build on top of in order to create new visualizations. In this subsection we briefly present the data standard and some of the more important structures built on top of it.

The standardized data format draws from the *three-table design for genomic data* [8] (**Sup. Fig. 1**). This design is capable of modelling the vast majority of data present in functional genomics experiments. Based on this design, we derived a data structure abstraction called, generically, *data source*. The *data source* acts as a table with

metadata annotating both rows and columns. Each row in a *data source* has a genomic coordinate, and each column corresponds to a measurement. Thus, each cell in the table represents the measurement value at a particular coordinate. *Data source* tables tend to be large enough for it not to be feasible to store them entirely in memory. For this reason, Epiviz retrieves only chunks of these tables in memory as needed. *Data sources* are treated as single tables in the Epiviz logic; however in practice, their corresponding data often comes from different physical tables or even different data providers. For this reason, coordinate information, common to all measurements in a data source, is separated from the actual measurements and retrieved independently, so redundant operations are avoided and no more data than needed is loaded in memory at any given time.

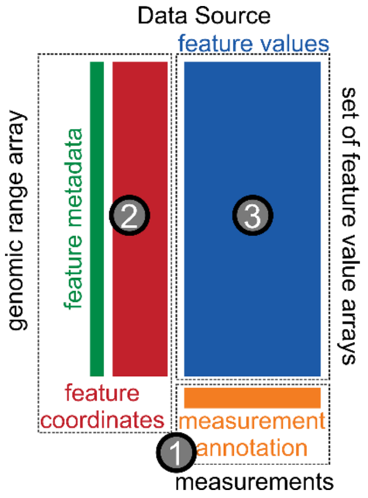

Data Source Example

Feature metadata (row annotation)

Affymetrix probe id	Gene symbol
1560477_a_at	SAMD11
202115_s_at	NOC2L
1555650_at	KLHL17
229792_at	KLHL17
224125_at	PLEKHN1
224501_at	C1orf170
227347_x_at	HES4
205483_s_at	ISG15

Feature coordinates

Global index	Sequence name	Start	End
1	chr1	861118	879955
2	chr1	879584	894670
3	chr1	895967	901095
4	chr1	895967	901095
5	chr1	901877	910488
6	chr1	910579	917497
7	chr1	934342	935552
8	chr1	948803	949920

Feature values

S259617	S259618
0.044001743	0.181882678
0.919555134	1.527183872
-0.467737552	-0.692543664
-0.230478323	-0.793671654
0.129586962	-0.24287666
0.951870488	-0.544007123
1.166289463	2.18030808
5.443433565	6.44870428

Measurement (sample) annotation

Tissue type	colon	colon
Subtype	normal	tumor

Sup. Fig. 1. Epiviz Data Sources. A standardized data format based on the *three-table* design. The left schematic shows the general structure, while the right one shows an example for microarray gene expression data. The three tables are 1) *measurements*, 2) *feature coordinates* stored as *genomic range arrays*, and 3) *feature values*, stored as sets of *feature value arrays*. Each data point is a *measurement* of a particular *feature* from a specific *sample. Features* occupy a *coordinate space* and have associated metadata. Samples have annotations associated as well.

An important substructure of *data sources* is constituted by *genomic arrays*. These encode all information corresponding to measurements' data objects. There are two types of genomic arrays in Epiviz – *genomic range arrays*, used to store coordinate information, and *feature value arrays*, used to store feature values for individual measurements. Genomic arrays are designed to store fragments of the data sets they represent, so that although not all data is loaded in memory at once, Epiviz modules can treat them as if they did. For this purpose, each row in a data source table has an identifier denoted as *global index*, which represents the index of a row in the entire data source, ordered by genomic coordinate. Global indices are passed in increasing consecutive order between modules, which simplifies operations on the UI that only require logarithmic time for searches and constant time for merging fragments of data.

Overview of performance optimizations

Epiviz implements a number of simple optimizations meant to improve user experience, which we discussed in our previous work, along with benchmarks underlining their effects over the overall user experience. These constitute our preliminary steps in this direction. In the following paragraphs, we expand over limitations and future research directions associated with this topic. The most important optimization consists of a predictive caching mechanism also depicted in **Sup. Fig. 2**. Due to this feature, the user only has to wait when opening Epiviz for the initial request to be fulfilled, most subsequent requests using the lag between user operations to load data likely to be requested on following actions. The other type of optimization is individual to each visualization, and consists of *binned aggregation*[9], also discussed in our previous work. In Chelaru et al.[10] we presented the extent of our current efforts in performance optimization.

Performance limitations

There are a number of performance limitations brought by the design choices of Epiviz. Some reflect the target use cases we built the software for. Others however, are issues we mean to address in future releases of the tool. In this section we underline some which we find more important.

First of all, the design of Epiviz restricts all optimization decisions to those that can be done on the client side. This is because our software, apart from a small server component that stores user data and workspaces information, runs entirely on the client machine, and proposes to aggregate data from any number of external sources. As we have no control over these data sources, optimization on the server side is not an option. This puts any complex data optimization techniques such as those discussed in [11] out of the scope of our current work.

Choosing JavaScript as the main programming language for the Epiviz framework comes with a number of advantages, but also a few limitations. The main advantage is that JavaScript runs natively in all major web browsers, on all operating systems, requiring no special installation. In addition, being optimized for online applications, it has a number of useful features that make it convenient to access online data sources. The fact that it is an interpreted language that can evaluate strings of text into executable code is also an important feature, which in Epiviz we make great use of. Finally, over the years, all major browsers have taken steps in isolating JavaScript, so that it is natively prevented from making changes to the local file system. This is essential for Epiviz in its intent to allow users incorporate third-party code to extend software functionality.

The main drawback of using JavaScript for Epiviz is its restriction to running into a single thread of execution. Although HTML5 introduces web workers[12] as an alternative to multi-threaded computing, in practice, because they are treated as separate processes and do not share the context of the main program, they are hard to use for optimizations closely related to the visualizations. For this consideration, operations on relatively large amounts of data tend to impact the performance of Epiviz and lower user experience. Workspaces with a large amount of visualizations, each showing a large number of data objects present latencies in responsiveness, as all objects in all visualizations are drawn using a single processing thread.

Another design decision that adds to the performance overhead is the use of vector graphics (SVG) for rendering visualizations. This is essential for some of the most important Epiviz features, such as brushing, tooltips, etc. At the same time, vectorial displays do not benefit from the limitation over the number of objects on the screen corresponding to the number of pixels available in the screen resolution, which comes with raster images. This means that the actual number of objects rendered on the screen will be proportional to the number of data records behind the visualization. In other words, even when objects overlap, each of them is still rendered and has a corresponding element in the HTML document. This, combined with the limitation of single-thread processing in JavaScript, leads to performance hits when a lot of visualizations with many objects are in view. In addition, because of the diverse nature of visualizations, it is impossible to predict which records will correspond to overlapping visual objects at the visualization API level, which moves the burden of visual optimization to each individual chart.

In the *Future research directions* section, we expand on ways in which we plan to address these performance limitations in future versions of Epiviz.

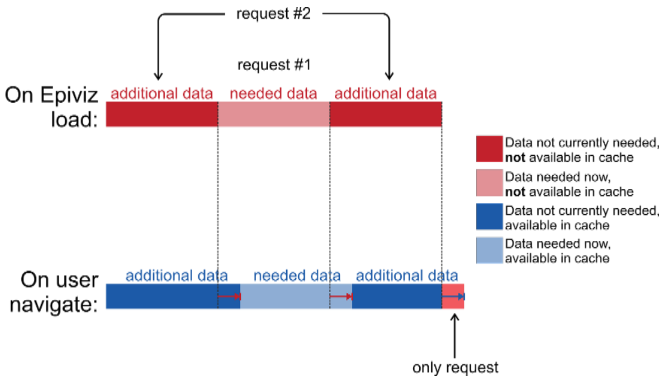

Sup. Fig. 2. The Epiviz predictive caching mechanism. On load or navigation to a new region in the genome, the data manager makes two requests: first for the data needed to update the visualizations on screen, and second for data in its vicinity. Once this data is loaded, subsequent pan and zoom operations are executed immediately, as all data necessary to fulfill them is already stored in memory. These subsequent operations also trigger a small additional request per operation, to keep the data in the cache consistent.

Software security through code sanitization

The Caja library establishes a connection to a Caja server hosted at http://caja.appspot.com. When users of Epiviz supply custom code, by using JavaScript dynamic extension, our framework asks Caja to construct a virtual DOM to which objects in the script are limited to. Epiviz also supplies a series of *defensive objects* to Caja. *Defensive objects* are the only objects within the framework that are accessible to the script and their implementation assumes that their clients *can* be malicious. Caja transforms the code to make it safe to run, by sending a GET request to the Caja server, wrapping the raw user code. The server returns the transformed code. The creators of the library call this process *cajoling*. Cajoling involves adding inline checks to make sure the code does not break the invariants Caja needs, and ensuring that the code cannot refer to variables in the host page that are not explicitly given to it. It also makes sure that the user code only uses the API published by the Epiviz framework.

From the viewpoint of the user code, it runs with what seems to be a W3C DOM compliant document object and an ECMAScript 5 compliant JavaScript virtual machine. Its document is confined to the boundaries of this virtual DOM, and its JavaScript global types and objects, like Object and Array, are its own and do not affect code outside it. The *defensive objects* are visible to user code as additional global variables in its top-level JavaScript context.

Using this library, Epiviz is able to restrict third-party scripts from any access to sensitive information such as the user cookie, opening pop-up windows or browser tabs, contacting third-party web servers, as well as the entire workspace data.

References

1. Zhou X, Maricque B, Xie M, Li D, Sundaram V, Martin EA, Koebbe BC, Nielsen C, Hirst M, Farnham P, Kuhn RM, Zhu J, Smirnov I, Kent WJ, Haussler D, Madden PAF, Costello JF, Wang T: **The Human Epigenome Browser at Washington University.** *Nat Methods* 2011, **8**:989– 90.

2. Goecks J, Nekrutenko A, Taylor J: **Galaxy: a comprehensive approach for supporting accessible, reproducible, and transparent computational research in the life sciences.** *Genome Biol* 2010, **11**:R86.

3. Goecks J, Coraor N, Nekrutenko A, Taylor J: **NGS analyses by visualization with Trackster.** *Nat Biotechnol* 2012, **30**:1036– 9.

4. Goecks J, Eberhard C, Too T, Nekrutenko A, Taylor J: **Web-based visual analysis for high-throughput genomics.** *BMC Genomics* 2013, **14**:397.

5. Cerami E, Gao J, Dogrusoz U, Gross BE, Sumer SO, Aksoy BA, Jacobsen A, Byrne CJ, Heuer ML, Larsson E, Antipin Y, Reva B, Goldberg AP, Sander C, Schultz N: **The cBio cancer genomics portal: an open platform for exploring multidimensional cancer genomics data.** *Cancer Discov* 2012, **2**:401– 4.

6. Green ED, Guyer MS: **Charting a course for genomic medicine from base pairs to bedside.** *Nature* 2011, **470**:204– 13.

7. **BD2K:NIH Big Data to Knowledge** [http://bd2k.nih.gov/#sthash.z6k1PpND.dpbs]

8. Gentleman RC, Carey VJ, Bates DM, Bolstad B, Dettling M, Dudoit S, Ellis B, Gautier L, Ge Y, Gentry J, Hornik K, Hothorn T, Huber W, Iacus S, Irizarry R, Leisch F, Li C, Maechler M, Rossini AJ, Sawitzki G, Smith C, Smyth G, Tierney L, Yang JYH, Zhang J: **Bioconductor: open software development for computational biology and bioinformatics.** *Genome Biol* 2004, **5**:R80.

9. Liu Z, Jiang B, Heer J: **ImMens: Real-time visual querying of big data**. *Comput Graph Forum* 2013, **32**:421– 430.

10. Chelaru F, Smith L, Goldstein N, Bravo HC: **Epiviz: interactive visual analytics for functional genomics data**. *Nat Methods* 2014, **11**:938– 940.

11. Lins L, Klosowski JT, Scheidegger C: **Nanocubes for real-time exploration of spatiotemporal datasets**. *IEEE Trans Vis Comput Graph* 2013, **19**(August):2456– 2465.

12. **HTML5 Web Workers** [http://www.w3schools.com/html/html5˙webworkers.asp]

13. **A client for retrieving Bioconductor objects from AnnotationHub** [http://www.bioconductor.org/packages/release/bioc/html/AnnotationHub.html]

14. **Using the BioSeqClass Package** [http://www.bioconductor.org/packages/release/bioc/vignettes/BioSeqClass/inst/doc/BioSeqClass.pdf]

15. **RDRToolbox: A package for nonlinear dimension reduction with Isomap and** [http://www.bioconductor.org/packages/release/bioc/vignettes/RDRToolbox/inst/doc/vignette.pdf]

16. **Normalization: Bioconductor' s marray package** [http://www.bioconductor.org/packages/release/bioc/vignettes/marray/inst/doc/marrayNorm.pdf]

17. **Representation and Manipulation of Genomic Intervals** [http://bioconductor.org/packages/release/bioc/manuals/GenomicRanges/man/GenomicRanges.pdf]

18. Anders S, Pyl PT, Huber W: **HTSeq - A Python framework to work with high-throughput sequencing data.** *Bioinformatics* 2014.

19. Pedregosa F, Weiss R, Brucher M: **Scikit-learn: Machine Learning in Python**. *J Mach Learn Res* 2011, **12**:2825–2830.

20. Baber I, Tamby JP, Manoukis NC, Sangaré D, Doumbia S, Traoré SF, Maiga MS, Dembé lé D: **A python module to normalize microarray data by the quantile adjustment method.** *Infect Genet Evol* 2011, **11**:765–8.

Supplementary figures

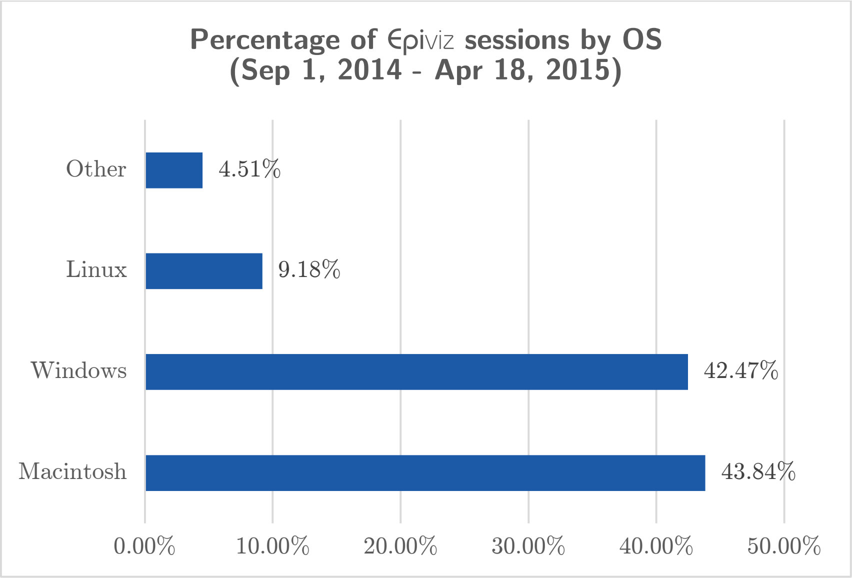

Sup. Fig. 3. Percentage of Epiviz sessions by OS since the time of its release, measured using Google Analytics.

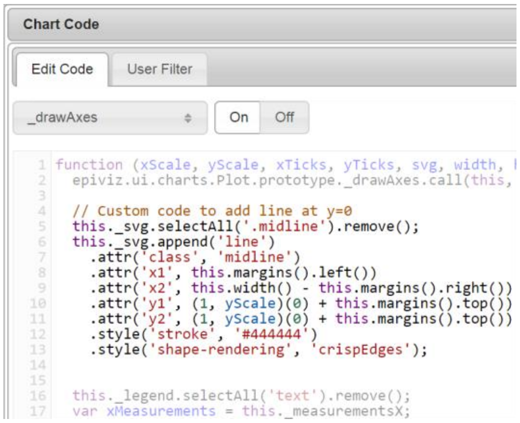

Sup. Fig. 4. The *Chart Code* dialog. Users of Epiviz can customize the code of individual visualizations directly in the UI, using this embedded JavaScript editor. In this example, we demonstrate the code necessary for adding a line at y=0 in the *scatter plot*.

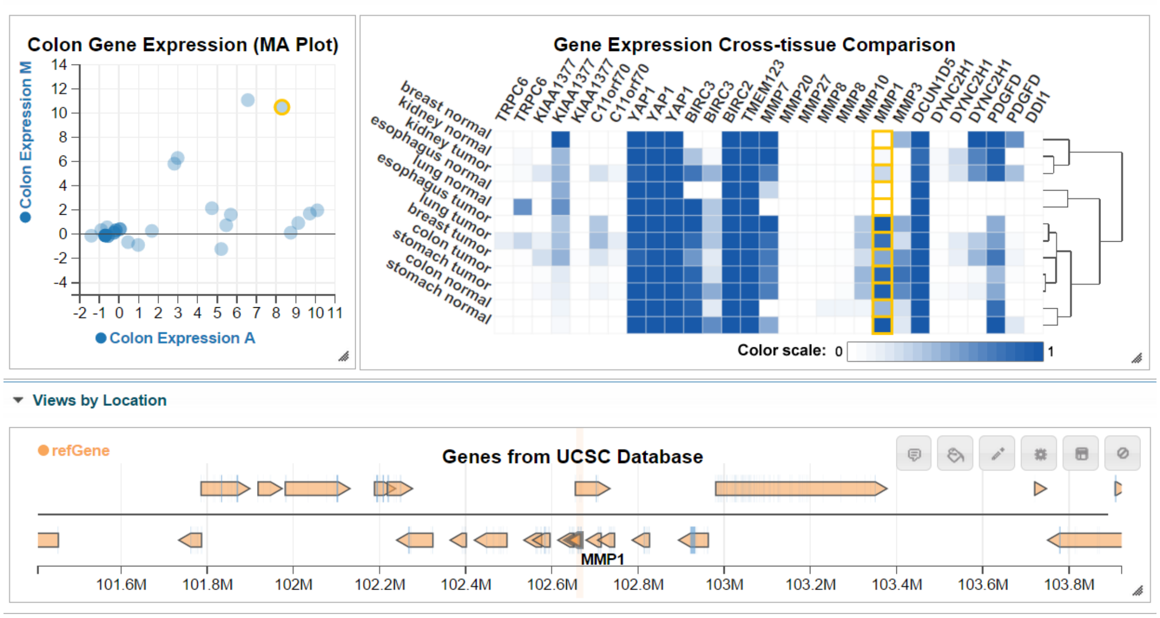

Sup. Fig. 5. A comparison of the gene expression of gene MMP1 across tissue types. Using the clustering feature of the *heatmap* we notice that the gene MMP1, along with MMP10 and MMP3 determine tumours to separate from healthy tissues, based on gene expression in this genomic region. http://epiviz.cbcb.umd.edu/?gist[]=5a88f39caa801e58b8ae&ws=S3ppS5e5cei

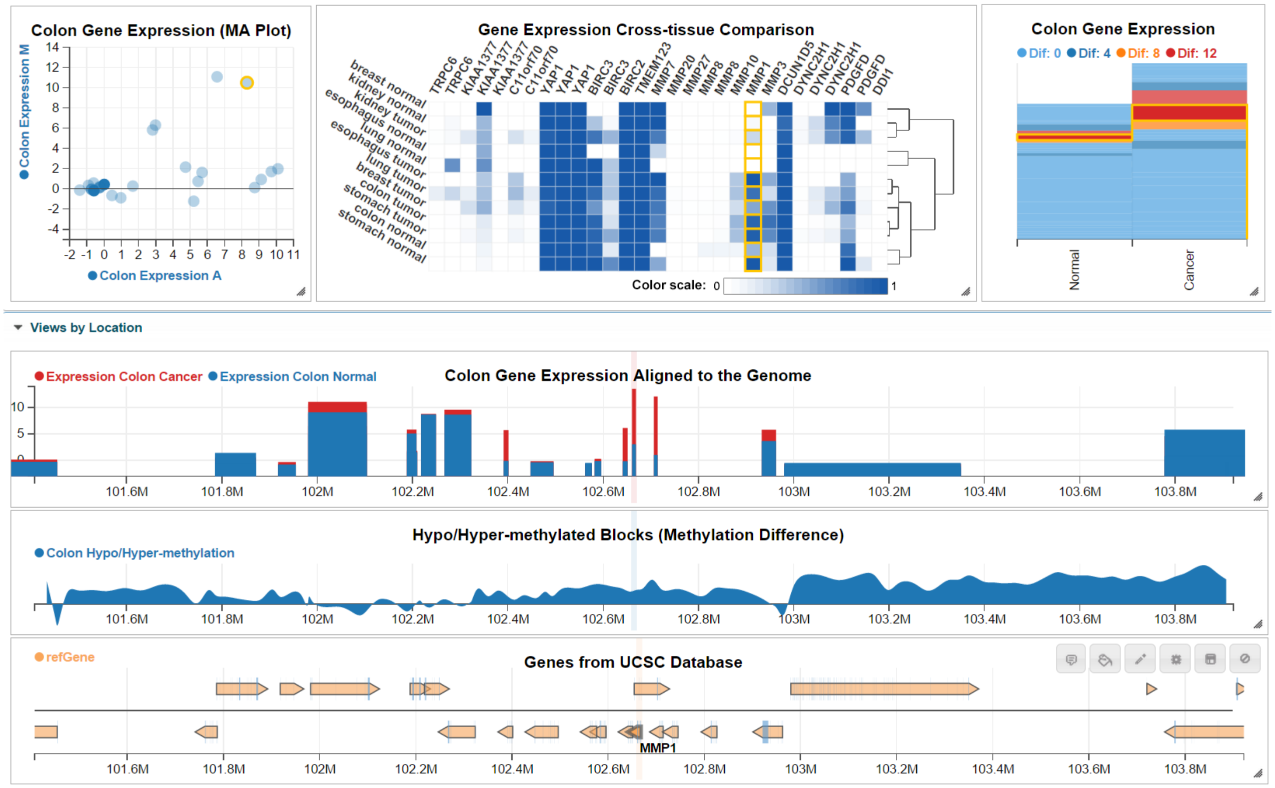

Sup. Fig. 6. Comparison of hypo/hyper-methylated regions and gene expression for normal and cancer colon tissues. To generate this figure, we created a visualization plugin stored on GitHub Gist (http://gist.github.com/160e8b84795603961b9f). The track shows blocks corresponding to colon gene expression values aligned to the genome, based on gene coordinates (first in the list of tracks in the figure). The second track is a stacked track showing a *computed measurement* corresponding to the difference in methylation levels between normal and cancer tissues.

http://epiviz.cbcb.umd.edu/?gist[]=160e8b84795603961b9f&gist[]=5a88f39caa801e58b8ae&ws=u4woieRRaxp

Supplementary tables

SUP. TABLE 1
PARTIAL LIST OF GENOMIC DATA ANALYSIS TASKS

Analysis Step	*Common Tasks at this Step*	*Epiviz Support*
Algorithmic Analysis (Data Modeling)	- simple data transformation	- through *computed measurements*<br>- through *UI code data transformations*
	- searching	- through the *gene search* feature
	- normalization, aggregation, smoothing, filtering, sorting, mapping, overlapping, dimensionality reduction, feature extraction	- in R/Bioconductor via Epivizr [13], [14], [15], [16], [17]<br>- in Python via Epivizpy[18], [19], [20]
Interactive Visualization (Exploration, Validation and Presentation)	- overview (low res), detailed view (high res), navigate, side-by-side view for comparison of multiple types of data, different views for the same data in different dimensions	- multi-view, pan/zoom
	- jump between regions of interest	- Epivizr's *slideshow* feature
	- linking between data sets using metadata or genomic location	- brushing
	- use visualizations for presentation (paper figures, etc.)	- *save as static image* feature<br>- workspace sharing

SUP. TABLE 2
PARTIAL LIST OF BIOCONDUCTOR DATA TYPES

Object type	*Data Standard*	*Bioconductor Interface*	*Bioconductor Class*
Sequence Data	FASTA	*FaFile*	*DNAStringSet*
Genomic Intervals	BED/bigBED	*BEDFile*	*GenomicRanges*
Genomic quantitative data	WIG/bigWIG	*BigWigFile*	*RleList*
ShortRead Alignments	SAM/BAM	*BamFile*	*GenomicAlignments*
Genomic Variants	VCF	*VCF*	*VRanges*

Through this set of data types, AnnotationHub and Epivizr provide integration of a variety of data through uniform data types, based on community standards, regardless of data source.
